# Prognostic value of body composition out of PSMA-PET/CT in prostate cancer patients undergoing PSMA-therapy

**DOI:** 10.1007/s00259-025-07416-7

**Published:** 2025-06-28

**Authors:** Wolfgang Roll, Lucas Plagwitz, David Ventura, Max Masthoff, Clemens Backhaus, Julian Varghese, Kambiz Rahbar, Philipp Schindler

**Affiliations:** 1 Department of Nuclear Medicine, University Hospital Münster, Münster, Germany; 2 West German Cancer Centre, Münster, Germany; 3 Institute of Medical Informatics, University of Münster, Münster, Germany; 4 Department of Radiology, University Hospital Münster, Münster, Germany

**Keywords:** PSMA-therapy, Body composition, PSMA-PET

## Abstract

**Background:**

This retrospective study aims to develop a deep learning-based approach to whole-body CT segmentation out of standard PSMA-PET-CT to assess body composition in metastatic castration resistant prostate cancer (mCRPC) patients prior to [^177^Lu]Lu-PSMA radioligand therapy (RLT). Our goal is to go beyond standard PSMA-PET-based pretherapeutic assessment and identify additional body composition metrics out of the CT-component, with potential prognostic value.

**Methods:**

We used a deep learning segmentation model to perform fully automated segmentation of different tissue compartments, including visceral- (VAT), subcutaneous- (SAT), intra/intermuscular- adipose tissue (IMAT) from [^68^ Ga]Ga-PSMA-PET-CT scans of *n* = 86 prostate cancer patients before RLT. The proportions of different adipose tissue compartments to total adipose tissue (TAT) assessed on a 3D CT-volume of the abdomen or on a 2D single slice basis (centered at third lumbal vertebra (L3)) were compared for their prognostic value. First, univariate and multivariate Cox proportional hazards regression analyses were performed. Subsequently, the subjects were dichotomized at the median tissue composition, and these subgroups were evaluated by Kaplan–Meier analysis with the log-rank test.

**Results:**

The automated segmentation model was useful for delineating different adipose tissue compartments and skeletal muscle across different patient anatomies. Analyses revealed significant correlations between lower SAT and higher IMAT ratios and poorer therapeutic outcomes in Cox regression analysis (SAT/TAT: *p* = 0.038; IMAT/TAT: *p* < 0.001) in the 3D model. In the single slice approach only IMAT/SAT was significantly associated with survival in Cox regression analysis (*p* < 0.001; SAT/TAT: *p* > 0.05). IMAT ratio remained an independent predictor of survival in multivariate analysis when including PSMA-PET and blood-based prognostic factors.

**Conclusions:**

In this proof-of-principle study the implementation of a deep learning-based whole-body analysis provides a robust and detailed CT-based assessment of body composition in mCRPC patients undergoing RLT. Potential prognostic parameters have to be corroborated in larger prospective datasets.

## Introduction

Prostate cancer (PC) is the second most common cancer of men worldwide [1]. The advanced stage of metastatic castration resistant prostate cancer (mCRPC) is associated with limited prognosis and survival [2]. Following standard androgen deprivation therapy, androgen receptor–targeted agents and chemotherapy [^177^Lu]Lu-PSMA therapy is an approved therapeutic option [3]. With an increasing demand for these radioligand therapies in mCRPC patients, patient selection is crucial considering other therapeutic options available and limited resources for PSMA-therapy.

PSMA-PET-CT is used as a gatekeeper for PSMA-therapy offering important information on the receptor status in metastatic disease [4]. Parameters derived from PSMA-PET-CT show predictive and prognostic value in retrospective analysis with partly contradictory results, e.g. regarding tumor volume [5–7]. In subanalysis of prospective vision study, however, only SUV_mean_ of all tumor lesions was a significant parameter with prognostic value (overall survival (OS)) in multivariate analysis [8].

Low Dose CT scans are routinely acquired during PSMA-PET-CT for attenuation correction and anatomic correlation purposes. However, these additional datasets are not routinely used for response assessment or prediction in prostate cancer patients. Body composition parameters can be easily assessed on the level of the third and fourth lumbar vertebrae (L3–L4) by either manual or (semi-) automatic contouring. From CT-studies it is well known that body composition parameter as visceral/subcutaneous fat or sarcopenia are associated with worse outcomes in mCRPC patients [9–11]. Previously, Hartrampf et al. could not demonstrate a prognostic value of body composition parameters, assessed on L3-L4 level, in patients undergoing PSMA-therapy [12]. But increased fat reserves as assessed by laboratory parameters were associated with improved OS [12].

We therefore aimed to assess body composition parameters using the a 3D approach, potentially overcoming limitations of 2D single slice approaches, by using a previously published deep learning-based segmentation framework. First data from other cancer entities suggest a prognostic role of deep learning-based 3D assessment of tissue volumes [13–15]. Our goal is to go beyond standard PSMA-PET-based pretherapeutic assessment and identify additional whole body composition metrics out of the routinely acquired low-dose CT-component, with potential prognostic value.

## Materials and methods

### Patients and study protocol

A total of 86 patients recommended for PSMA therapy by an internal tumor board were enrolled in this retrospective study. All patients satisfied the following inclusion criteria adapted from VISION-study [3]: (a) mCRPC with PSMA-positive disease (uptake greater than liver, no relevant PSMA-negative metastases), (b) disease progression after one or more approved androgen deprivation therapies and at least one taxane-based chemotherapy, (c) a life expectancy of at least six months, (d) an Eastern Cooperative Oncology Group performance status ≤ 2, and (e) adequate organ function and bone marrow reserve. Written informed consent was obtained from all patients for both PSMA-PET-CT and radioligand therapy. This study was approved by the local ethics committee (No. 2016–585-f-S and further amendments, Ethikkommission der Ärztekammer Westfalen-Lippe und der Universität Münster).

### PSMA-PET

PSMA-PET-CT was performed following institutional protocols. Before the examination patients signed written informed consent. A median of 150 MBq [^68^ Ga|Ga-PSMA-11 was applied intravenously and the patient was hydrated with normal saline afterwards.

A whole-body scan from vertex to proximal femur was performed 60 min post injection using a Siemens Biograph mCT 128 System (Siemens Healthcare, Erlangen, Germany). For attenuation correction a low-dose-CT was performed, using the following specification: 120 kVp, 30 mAs (CareDose4D modulation), pitch of 1.2, collimation of 0.6 mm, and reconstructed slice thickness of 1.5 mm with a B31f kernel. This low dose CT was used for further body composition analysis. PSMA-PET based parameters assessed, include (1) the PSMA positive tumor volume (50% of local SUV_max_) and (2) the mean SUV_mean_ of the PSMA-PET positive tumor volume.

### Body segmentation

We employed the open-source CT image segmentation algorithm, body and organ analysis (BOA) [16–18], to facilitate our body composition analysis. The automatically generated report structure of the tool was utilized for this study. In this regard, we evaluated the following outputs: total adipose tissue (TAT), intramuscular adipose tissue (IMAT), subcutaneous adipose tissue (SAT), and visceral adipose tissue (VAT). These measurements were examined in two configurations: (1) The total sum of the parameters across the torso, excluding the extremities (volumetric/3D approach), (2) a focused evaluation of the centrally located L3 slice. Pericardial (PAT) and epicardial (EAT) adipose tissue was assessed in the volumetric approach only due their constant zero values in L3-slice region.

For further analyses, measurements of TAT were reported in millilitres (mL). IMAT, SAT, VAT, PAT and EAT were expressed as proportion of TAT. The expression as ratios of TAT allows for correction of slight differences in scan volume and stature and were therefore given preference over absolute volumes.

### Statistical analysis

All data were analyzed using Python, leveraging specific packages such as lifelines and statsmodels [19, 20]. Initially, the continuous measurements were assessed using univariable Cox proportional hazards regression. For subsequent multivariable Cox regression model, a univariable inclusion criterion of *p* ≤ 0.2 was used. To compare model quality between the L3-slice and volume-based approaches, we assessed partial AIC, concordance index, and log-likelihood ratio. Fat-associated parameters that met the inclusion criteria in the multivariate Cox analysis were subsequently evaluated using univariate Kaplan–Meier (KM) analysis, followed by a log-rank test. Continuous variables were dichotomized at the median for KM analysis. All statistical tests had a level of significance established at *p* < 0.05.

## Results

### Patient characteristics

**Fig. 1:**
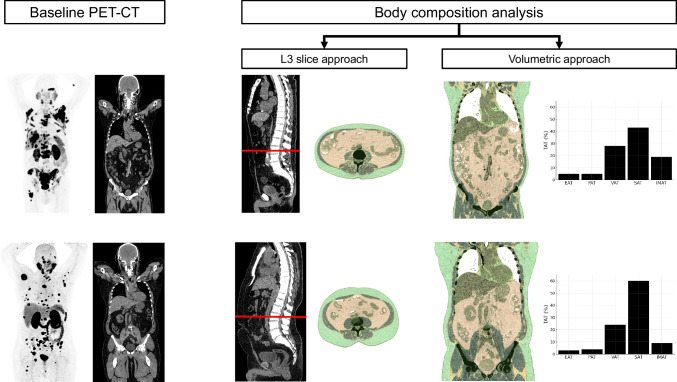
Automated segmentation workflow, including the L3-based- and volumetric assessment of different adipose tissue fractions out of the low-dose-CT component of a PSMA-PET/CT of a patient with lower SAT/TAT- and higher IMAT/TAT-ratio (upper row) compared to another patient with higher SAT/TAT- and lower IMAT/TAT-ratio (lower row). IMAT: intramuscular adipose tissue, L3: Third lumbar vertebra, SAT: Subcutaneous adipose tissue, TAT: total adipose tissue

A total of 86 patients who met all inclusion criteria were included in this study (Fig. 1). An overview of patient characteristics is presented in Table 1.

**Table 1:** Patient characteristics of 86 Patients

Characteristic		*n*	%	median	range
Men		86			
Age				70	44—87
ISUP Grade					
Grade 1		6	7.0		
Grade 2/3		24	27.9		
Grade 4		22	25.6		
Grade 5		34	39.5		
Pretreatments					
Primary local treatment		58	67.4		
Chemotherapy		71	82.6		
Androgen receptor signaling inhibitors		86	100		
Disease localization					
Lymph node metastases		70	81.4		
Distant metastases		79	91.5		
	Bone	78	90.7		
	Liver	20	23.3		
	Lung	16	18.6		
	Brain	1	1.2		
ECOG* performance status					
ECOG 0		25	29.1		
ECOG 1		46	53.5		
ECOG 2		15	17.4		
Activity [GBq]				6.5	5.1—7.6
PSA* [ng/mL]				168.2	0.2—8403

*ECOG, Eastern cooperative oncology group performance status; *PSA*, prostate specific antigen

### Prognostic value of body composition analysis

The association between body composition parameters and OS was evaluated using univariate Cox proportional hazards regression, with results summarized in Tables 2 and 3. For both the L3-slice- and volume-based approach, parameters IMAT/TAT and SAT/TAT met the criteria of multivariate inclusion (*p* < 0.2) with lower IMAT/TAT and higher SAT/TAT being associated with improved survival. In multivariate analysis, IMAT/TAT remained an independent predictor of survival for both approaches (single slice, *p* = 0.023; 3D volume, *p* = 0.015), while SAT/TAT was not significant. PAT/TAT and EAT/TAT, not included in L3 slice assessment, do not provide additional prognostic value in Cox proportional hazard analyses including 3D body composition parameters. As mentioned above, we specified the absolute volumes in relation to the TAT for normalization purposes. Moreover, other potentially prognostic parameters were included into uni- and multivariate analysis, namely age, PSA-value, haemoglobin, LDH and PSMA-PET total SUV_mean_ and PSMA-tumor volume (PSMA-TV). LDH, SUV_mean_ and PSMA-TV were associated with survival in univariate analysis. In multivariate models SUV_mean_ remained significant including L3-level body composition parameters, however not within the analysis of 3D IMAT/TAT.

**Table 2:** Univariate and multivariate Cox proportional hazard analyses of body composition assessed on L3 level for overall survival (*n* = 86)

	Univariate Analysis				Multivariate Analysis			
Part	HR	lower 95%	upper 95%	*p*	HR	lower 95%	upper 95%	*p*
Age	1.020	0.975	1.067	0.392				
PSA	1.0	1.0	1.0	0.228				
Hb	0.982	0.948	1.018	0.325				
LDH	1.002	1.0	1.004	**0.022**	1.001	0.999	1.004	0.228
PSMA-TV	1.003	1.001	1.005	**0.110**	1.002	1.000	1.005	0.088
PSMA-SUV_mean_	0.808	0.671	0.973	**0.025**	0.749	0.593	0.896	**0.041**
Muscle	1.009	0.945	1.079	0.773				
TAT	0.998	0.988	1.009	0.736				
IMAT/(TAT)	1.155	1.108	1.239	** < 0.001**	1.130	1.050	1.221	**0.023**
SAT/(TAT)	0.981	0.954	1.008	**0.160**	0.100	0.002	6.040	0.271
VAT/(TAT)	1.002	0.977	1,028	0.853

**Table 3:** Univariate and multivariate Cox proportional hazard analyses of body composition volumes (3D) for overall survival (*n* = 86)

	Univariate Analysis				Multivariate Analysis			
Part	HR	lower 95%	upper 95%	*p*	HR	lower 95%	upper 95%	*p*
Age	1.020	0.975	1.067	0.392				
PSA	1.0	1.0	1.0	0.228				
Hb	0.982	0.948	1.018	0.325				
LDH	1.002	1.0	1.004	**0.022**	1.002	0.999	1.005	0.138
PSMA-TV	1.003	1.001	1.005	**0.110**	1.003	1.001	1.005	0.617
PSMA-SUV_mean_	0.808	0.671	0.973	**0.025**	0.988	0.955	1.023	0.507
Muscle	1.022	0.811	1.288	0.851				
TAT	1.000	0.964	1.041	0.997				
IMAT/(TAT)	1.204	1.115	1.300	** < 0.001**	1.316	1.072	1.639	**0.015**
SAT/(TAT)	0.961	0.925	0.998	**0.038**	0.052	0	10.031	0.271
VAT/(TAT)	1.003	0.965	1.043	0.881				
PAT/(TAT)	0.86	0.360	2.055	0.734				
EAT/(TAT)	1.96	0.647	5.935	0.234				

The two multivariate Cox proportional hazards models (L3-slice vs volume) were compared to assess the impact of the variables on model performance. The volume-based model had a lower AIC (195.6 vs. 200.7) and a higher Harrell's C-index (0.67 vs. 0.58) indicating a better fit and slightly higher predictive accuracy. Likelihood-ratio testing using a union model confirmed that inclusion of volume-based IMAT/(TAT) significantly enhanced model fit over the L3-slice model (χ^2^ = 6.72, *p* = 0.009).

### Body-composition based survival analysis

**Fig. 2:**
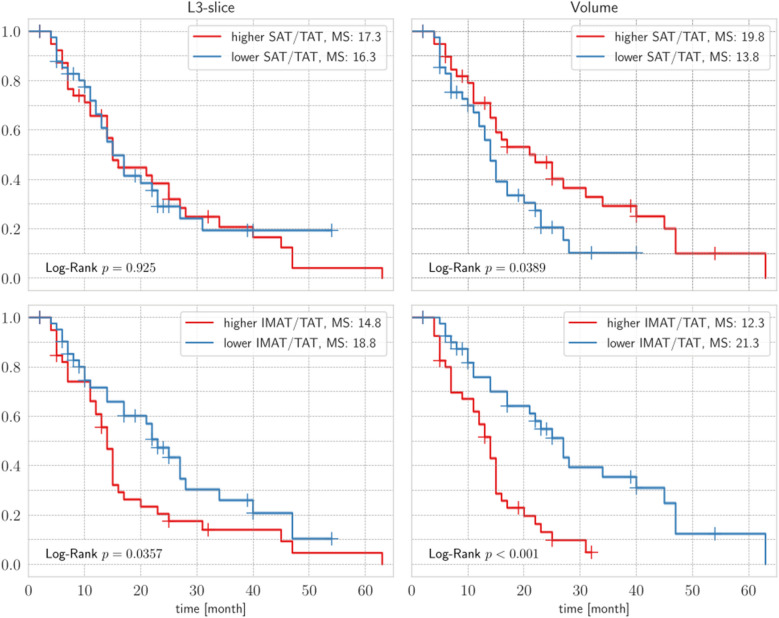
Overall survival of patients with prostate cancer based on SAT/TAT ratio and IMAT/TAT ratio measured compared between L3-slice and abdominal cavity volume. MS: Mean Survival, IMAT: intramuscular adipose tissue, SAT: Subcutaneous adipose tissue, TAT: total adipose tissue

We additionally conducted a Kaplan–Meier survival analysis, followed by a Log-Rank test, to compare L3-slice and volume-based IMAT/TAT and SAT/TAT ratios. Groups were divided at the median. Figure 2 displays all four subplots. The SAT/TAT ratio based on the L3-slice showed no significant difference, with a mean survival of approximately 17 months in both groups (Log-Rank *p* = 0.88). In contrast, the volume-based SAT/TAT ratio indicated a modest difference, revealing improved OS in the group with a higher SAT/TAT ratio (mean: 19.79 vs. 13.84 months, Log-Rank *p* = 0.042). For IMAT/TAT, the L3-slice approach demonstrated shorter survival associated with a higher IMAT/TAT ratio (mean: 14.84 vs. 18.79, Log-Rank *p* = 0.05). In the multivariate analysis, this exhibited a strong correlation with OS (mean: 12.35 vs. 21.28, Log-Rank *p* < 0.001).

## Discussion

In this retrospective proof-of-concept study, a fully automated, deep learning–based body composition analysis of the different fat compartments from the CT component of PSMA-PET/CT scans provides additional prognostic information in mCRPC patients undergoing PSMA therapy. Our findings suggest that volumetric measures of IMAT and SAT normalized by TAT, correlate with OS. Volumetric measures outperformed L3 slice–based semiautomatic assessment, allowing for a more comprehensive evaluation of body composition and improved predictive power in multivariate models.

Standard body composition assessment at the level of the L3 vertebra, either semiautomatically or manually, has been extensively studied in various malignancies [13, 14, 21]. Prior investigations into the prognostic role of body composition in prostate cancer have yielded mixed results. Several studies have linked increased visceral adipose tissue, overall adiposity, or sarcopenia with poorer outcomes [9, 11, 21–23]. In contrast Hartrampf et al. reported no prognostic value for body composition parameters derived from L3–L4 level in patients receiving PSMA-directed radioligand therapy [12]. However, such a two-dimensional approach does not necessarily accurately reflect the heterogeneity of different tissue compartments in mCRPC patients. Adopting a three-dimensional analysis provides more accurate assessment of adipose tissue fractions [24]. These volumetric compartments add additional discriminatory power for outcome prediction, outperforming a 2D slice based assessment [25, 26]. Decazes et al. reported on improved prognostication of CT-based 3D adipose tissue compartments in correlation to OS in patients receiving anti-angiogenic drugs [26]. Jung et al. used a deep learning analysis of body composition parameters out of whole-body MRI. The association between body composition parameters and survival was attenuated for the slice-based assessment compared to 3D volumetric assessment [25]. This is in line with the results from our study with SAT/TAT showing a modest yet statistically significant prognostic association with OS in the 3D model only. The volumetric model also showed better performance metrics for IMAT compared to the L3-assessment. Another possible explanation for discrepancies lies in the fully automated deep learning pipeline, which mitigates operator-related variability and might capture more comprehensive information about the patient’s body composition compared to manual or semiautomatic approaches [25]. The 3D model offers the possibility to assess parameters not included in the L3-level slice-based assessment, as PAT and EAT, with potential prognostic value, especially in cardiovascular diseases [27]. However, these parameters did not provide additional value for predicting response in patients undergoing [177Lu]Lu-PSMA radioligand therapy in this study.

Interestingly, neither VAT/TAT nor skeletal muscle measures emerged as significant prognostic factors in our cohort. Although VAT is often regarded as a potential prognosticator for adverse outcomes, conflicting reports exist regarding its importance in advanced prostate cancer [21, 23, 28]. In a Metaanalysis by Lopez et al. SAT and VAT/SAT, however not VAT alone, provided a significant prognostic value in prostate cancer patients [28]. However, most of the studies included assessed body composition parameters on L3/L4 level only [28]. Recently a large database analysis by Jung et al. in 36317 patients from the UK Biobank revealed that skeletal muscle, skeletal muscle fat fraction and IMAT were independent prognosticators for mortality [25]. Similarly in our study IMAT/TAT is the strongest predictor for OS, also in multivariate analysis.

IMAT is known to be inversely correlated with muscle strengths being a parameter for muscle quality [29]. Moreover, IMAT was an independent marker, independent of muscle mass, for limited physical performance and mobility [29, 30]. Inflammation as a main driver of the development from obesity to sarcopenic obesity results in increasing IMAT followed by muscle loss [29]. A potential confounder limiting comparability of previous studies and ours is the different stage of the disease, potentially influencing body composition parameters.

Many studies have focused on a potential predictive or prognostic value of the PET component of PSMA-PET-CT [7, 31]. In line with the results from our cohort, out of all PET-based parameters, whole body tumor SUV_mean_ was the strongest predictor of response, and prognosticator for PFS and OS in patients undergoing [177Lu]Lu-PSMA radioligand therapy in prospective VISION study[32]. Different blood-based parameters, as LDH with potentially predictive value, are included in nomograms for a more accurate prediction of OS and PFS[33]. In our study, body composition parameters even outperformed blood-based parameters, however in a relatively small and retrospective cohort. By leveraging the routinely acquired CT component, clinicians have a unique opportunity to derive body composition metrics at essentially no additional radiation cost or procedural burden. Using all available information from the PET and CT component [34, 35], potentially including additionally acquired MRI [36], might augment selection criteria for RLT and informing clinical decision-making. A promising future direction is to evaluate change in body composition parameters during therapy [22] to define early response compared to established parameters [37].

Several limitations must be acknowledged. First, the retrospective design of this study introduces potential biases in patient selection and data completeness. Second, our sample size of 86 patients, while sufficient to demonstrate proof-of-concept, may constrain the generalizability of our results. Third, we focused on a single-center population undergoing RLT, possibly limiting broader applicability. Additional research should validate these findings in larger, multicenter, prospective trials, ideally with standardized protocols for imaging acquisition and analysis. This includes standardization of methods of normalization. Currently a wide variety of predefined volumes or ratios to other body/tissue volumes is used in different studies [13, 25, 26, 29]. Extracting body composition parameters, bone is a complicated marker in prostate cancer, due to the highly frequent presence of bone metastases in mCRPC and the influence of androgen deprivation therapy on bone composition [38]. We have therefore decided to exclude bone from our analysis and have focused on the different adipose tissue compartments.

In conclusion, we present an automated, deep learning–based methodology for whole-body CT segmentation that adds significant value to the prognostic assessment of mCRPC patients treated with [177Lu]Lu-PSMA radioligand therapy. The volumetric evaluation of IMAT and SAT, particularly in relation to TAT, appears to identify patients at higher risk of inferior survival outcomes. These results warrant further confirmation in prospective settings and suggest an evolving role for quantitative body composition analysis as a valuable adjunct to established PSMA-PET imaging markers.

## Data Availability

The datasets used and/or analysed during the current study are available from the corresponding author on reasonable request.
